# Editorial: Structural and Functional Characterization of Circular RNAs

**DOI:** 10.3389/fmolb.2021.795286

**Published:** 2021-11-02

**Authors:** Ioannis Grammatikakis, Florian A. Karreth, Amaresh C. Panda

**Affiliations:** ^1^ National Cancer Institute, National Institutes of Health (NIH), Bethesda, MD, United States; ^2^ H. Lee Moffitt Cancer Center and Research Institute, Tampa, FL, United States; ^3^ Institute of Life Sciences, Bhubaneswar, India

**Keywords:** circular RNA, competing endogenons RNA network, microRNA, RNA-bindig proteins, gene regulaiton

Circular RNAs (circRNAs) are a class of covalently closed RNA molecules without any free ends. The existence of circRNAs was first discovered in plant viroids, followed by their discovery in eukaryotic cells by electron microscopy more than 40 years ago (Sanger et al., 1976; Hsu and Coca-Prados, 1979). However, they were mostly considered non-functional splicing byproducts or intronic lariats. Their identification and functional characterization progressed slowly until the development of high-throughput RNA-sequencing methods and novel computational tools (Salzman et al., 2012; Jeck et al., 2013). The last few years have seen an exploding number of studies elucidating the molecular mechanisms of biogenesis and function of circRNAs. Recent discoveries established that circRNAs are generated by the backsplicing of pre-mRNA, mediated by RNA-binding proteins (RBPs) and inverted repeat sequences in the flanking introns (Jeck et al., 2013; Chen and Yang, 2015). Interestingly, circRNAs have been identified in all eukaryotic model organisms and are believed to be ubiquitously expressed, and some of the circRNAs are conserved across species (Jeck et al., 2013). Moreover, the altered expression of circRNAs during development and diseases has been reported, and their extraordinary stability and presence in biofluids make them promising biomarkers for disease diagnosis (Zhang et al., 2018). Increasing evidence suggests that circRNAs regulate the activity of microRNAs and RNA-binding proteins, and some are translated into peptides (Panda et al., 2017; Panda, 2018; Sinha et al., 2021). Recent studies have shown that circRNAs play critical roles in various pathophysiological processes (Lee et al., 2019). Although circRNAs have been established as crucial regulators of gene expression and disease development, many of the molecular details of circRNA biogenesis and function remain to be explored. In addition, only a tiny subset of circRNAs has been functionally characterized among more than a million circRNAs identified thus far (Vromman et al., 2021). Based on the above, this research topic aimed at contributing towards the elucidation of the role of circRNAs in various physiological and pathological conditions.

The review articles in this topic were all directed towards discussing the recent progress in the field of circRNA biology and their relevance in human health. Guria et al. provide an extensive overview of the mechanisms of circRNA biogenesis, classification and nomenclature, methods of detection, and their role in animal and plant physiology. Zhang et al. summarize the expression and regulatory role of circRNAs in plant growth, development, and stress responses. Qin et al. highlight the current knowledge of structure, biogenesis, and function of linear lncRNAs and circRNAs. They also highlight the role of circRNAs as biomarkers for disease diagnosis. Another article by Li et al. discusses the current understanding of circRNA expression and function in the central nervous system. Acha et al. review the current knowledge of circRNAs in blood malignancies and their potential value as diagnostic and therapeutic targets. Rajappa et al. discuss the emerging potential of circRNAs in cancer diagnosis and therapy. Another article by Liu et al. reviews the current knowledge of circRNAs in cervical cancer and the strategies for the use of circRNAs in future clinical diagnosis, prognosis, and treatment of cervical cancer. Zucko and Boris-Lawrie highlight the current understanding of circRNAs and the use of translational outcomes of circRNA research to improve the health and productivity of food animals.

The original research articles in this topic expand our understanding of the physiological significance of circRNAs in disease development. Using computational analyses, Khan et al. provide a comprehensive mechanism of gene regulation through circRNA–miRNA–mRNA regulatory networks in various cancers. Li et al. present data demonstrating that *circTLK1* promotes glioma progression by activating JAK/STAT signaling through the miR-452-5p/SSR1 pathway. Liu et al. demonstrate that circGNB1 regulates cell proliferation, migration, and tumor growth in triple-negative breast cancer by regulating the miR-141-5p-IGF1R axis. Han et al. identified differentially expressed circRNAs in the peripheral blood samples of patients with heart failure compared to healthy humans. They also show that hsa_circ_0097435 could act as a sponge for various miRNAs and regulate myocardial cell injury. Zhang et al. identified thousands of circRNAs in the liver of Whitespotted Bamboo Shark and constructed the mRNA–miRNA–circRNA regulatory network for the Glutathione S-transferase P1 gene. Another interesting article by Sun et al. describes the presence of internal complementary base-pairing sequences in extremely long circRNAs, allowing the circRNAs to present in double-stranded or pseudoknot structures. They hypothesize the “open-close effect” which may be a novel molecular function of circRNAs.

In summary, this Research Topic expands our knowledge on the relevance of circRNAs in animal and plant physiology regulation. Also, it discusses the future use of current knowledge for the diagnosis and therapy of human diseases. We hereby thank all the authors for contributing to this exciting Research Topic.
